# What have we learnt from the past – would treatment decisions for GEP-NET patients differ between 2012 to 2016 by the new recommendations in 2022?

**DOI:** 10.1186/s12885-023-10567-1

**Published:** 2023-02-13

**Authors:** Rahel Stiefel, Kuno Lehmann, Thomas Winder, Alexander R. Siebenhüner

**Affiliations:** 1 Medical Oncology and Hematology, Triemli Hospital Zurich, Zurich, Switzerland; 2 Department of Surgery and Transplantation, University Hospital and University of Zurich, Zurich, Switzerland; 3 Internal Medicine II, Hematology, Oncology and Gastroenterology, Academic Teaching Hospital Feldkirch, Feldkirch, Austria; 4 University of Zurich, Zurich, Switzerland; 5 Department of Medical Oncology and Hematology, University Hospital and University of Zurich, Zurich, Switzerland; 6 Clinic of Internal Medicine and Oncology, Cantonal Hospital Schaffhausen, Schaffhausen, Switzerland

**Keywords:** GEP-NET, Treatment sequences, SSA, Overall survival, Progression free survival, Real-life population

## Abstract

**Background:**

Gastroenteropancreatic neuroendocrine tumors (GEP-NETs) are a heterogeneous group of tumors with a broad range of local and systemic treatment options. Still a lack of data regarding treatment sequences exists. The aim of this study was to analyse outcomes in GEP-NETs depending on stage and treatment steps and compare our treatment decisions to the latest treatment recommendations of European Society of Medical Oncology (ESMO) 2020 for GEP-NETs.

**Methods:**

Patients were included in this retrospective single-center analysis from 2012—2016. All patients suffering from a GEP-NET, who were screened, treated or evaluated at ENETS Center in Zurich, Switzerland were included in analysis. Patients with any other diagnosis of NET were not included. We used Kaplan Meier estimator as well as Cox regression to compare survival rates between different sites of localization, grades or stages and treatment sequences.

**Results:**

Overall, we identified 256 GEP-NETs, most in advanced stage (62%) and located in small intestine tract or pancreatic gland. Survival depended on stage, grade, primary site and duration of response for the early systemic treatment. On average patients underwent 2.6 different treatment modalities, mostly depending on stage and higher tumor grade. Surgery was performed early but also in advanced stages, usually followed by Somatostatine-Agonist modalities.

In distant disease (Stage IV), we investigated a positive effect of PFS after treatment with Somatostatine Analogues (SSA) (hazard ratio [HR], 0.45; 95% confidence interval [CI], 0.21 – 0.97; *p* = *0.04*) and systemic treatment (HR, 0.51; 95% CI, 0.26 – 0.99; *p* = *0.047*) if patients underwent prior surgery or endoscopic resection.

Kaplan Meier distributions predict shorter OS in distant disease (Stage IV), (Figure. 1; HR, 2.06; 95% CI, 1.46 – 2.89; log-rank test, *p* < *0.001*).

**Conclusion:**

This retrospective analysis presents a great overview of all patients’, disease and treatment characteristics of GEP-NETs at ENETS Center in Zurich, Switzerland. We illustrated survival (PFS) depending on implemented therapies. According to these findings, we formed a suggested treatment algorithm for advanced GEP-NETs, which does not differ from the latest treatment recommendation by ESMO guidelines for GEP-NETs.

The results of this project may define GEP-NET patients’ selection for upcoming clinical prospective studies.

**Supplementary Information:**

The online version contains supplementary material available at 10.1186/s12885-023-10567-1.

## Introduction

Neuroendocrine tumors (NETs) are a rare and heterogeneous spectrum of tumors, originating from the embryonal neural crest. Gastroenteropancreatic (GEP)-NETs represent about 60% to 70% of all NETs and occur at an age-adjusted incidence of 5.25 cases per 100′000 people [1–3]. The annual report by SwissNET showed an increase by 26% between 2014 and 2015 and a stable increase of new NETs in Switzerland around 20% in 2020 [4, 5]. Although not fully understood, the rising incidence may also reflect better diagnostic tools or earlier detection, e.g. during appendectomy [6]. Apart from the primary tumor site, histological characteristics define and predict biological behaviour and prognosis of GEP-NETs [7–10].

According to the European Neuroendocrine Tumor Society (ENETS)/World Health Organization (WHO) grading system, NETs were until 2017 grouped into three grades according to tumor proliferation markers, including Ki-67 index and mitotic count (10 per high-power field [HPF]). This classification has moved forward in 2017, when the WHO grading system was updated and the group of NECs was subdivided into well-differentiated G3 NET (Ki-67 < 55%) and poorly differentiated NEC (Ki-67 index ≥ 55%) [8, 11, 12]. Another update of the WHO classification in 2019, highlighted a new terminology of mixed neuroendocrine-non-neuroendocrine tumors (MiNEN), formally classified as mixed adenoneuroendocrine carcinoma (MANEC) as well more detailed classifications of NET G3 and a terminology of appendiceal goblet cell carcinoma/carcinoid [13].

A precise staging is critical, as over 50% of GEP-NETs initially manifest as advanced or metastatic stage. In addition to metastases in lymph nodes, NET spreads predominantly into liver, peritoneum, bone and lung [14]. Different staging systems include the TNM staging systems of ENETS (mostly used in Europe) or American Joint Committee on Cancer/Union for International Cancer Control (AJCC/UICC, more used in the United States of America [USA]) [15, 16].

Treatment decisions for localized and advanced stages are made by a consensus of experts among a NET dedicated multidisciplinary tumor board (MTB) based on guidelines such as ENETS or ESMO for GEP-NETs [11, 17–23].

Curative treatment of GEP-NETs is only possible by surgical or endoscopic resection. This also refers to patients with limited (regional) disease or oligometastatic situation, whereas palliative surgery or ablation for symptom relief may also be considered in selected cases [24–26]. Over the last decades, treatment of metastatic NET has dramatically evolved, providing novel strategies. For example targeting the somatostatin-receptor by Somatostatin Analogues (SSA) is one of the best evaluated options [3]. Nowadays, long-acting Octreotide or Lanreotide are used to either control carcinoid symptoms by decreasing hormone and peptide secretion or control tumor growth and progression [27, 28]. These agents are mostly used for functional GEP-NETs or for management of well-differentiated proliferative tumors with expression of somatostatin receptors (SSTR) [29]. Other essential systemic therapies include chemotherapy combinations (Streptozotocin [STZ] and 5-fluorouracil [5-FU] or Capecitabine and Temozolomide [CAPTEM] in G2, or platin combination with Etoposide in G3 NETs). Recently, peptide receptor radionucleotide therapy (PRRT), using radiolabelled somatostatin analogues for targeting SSTR, enriched the treatment modality in second or further line [30]. Plus, targeting the Mechanistic Target of Rapamycin (mTOR) pathway or tyrosine kinase inhibitors (TKIs) in advanced pancreas-NETs (p-NETs), as well as Bevacizumab or interferon-alpha as back-up options has put more variety in the further-line treatment [3].

All these options are presented in the current guidelines of ENETS, ESMO or NCCN [11, 17–23, 31]. According to grading, staging and the origin of GEP-NET, these guidelines reflect a clear recommendation for first-line treatment. But no preference or sequence recommendation is clearly stated for GEP-NETs. While current treatment options have been approved by placebo controlled trials, a head-to-head comparison of active treatments is lacking [30, 32–36]. Thus, real-world data are highly valuable to contribute survival and treatment data, especially regarding the rarity of this disease [17, 20, 37].

Due to this wide range of systemic treatment possibilities, it is highly important to discuss a GEP-NETs situation among an interdisciplinary setting (if available at an certified ENETS Center of Excellence [CoE] [17, 38]. Therein, combined or sequential treatment options should be taken into consideration to improve survival [3, 11].

In this project we aimed to analyse differences in treatment and survival outcome of GEP-NET patients according to their course of treatments.

## Materials and methods

Between 2012 and 2016, patients with a diagnosis of GEP-NET were screened, treated or evaluated at the ENETS Center Zurich, Switzerland, including a follow-up until December 2018. Patients with any other diagnosis of NET were not included.

(Appendix A1) All patients’ data was retrospectively extracted from routinely documented medical history. At the University Hospital of Zurich, patient data including diagnostics and therapies were completely documented by electronic health record (EHR) and managed by a vendor EHR system (KISIM, CISTEC AG, Zurich, Switzerland). Missing medical reports of patients with lacking medical history files in our EHR system were requested at last attending physician. Patients’ and disease characteristics, such as kind of tumor and metastases, stages, histologic grades and dates of progression; diagnostics, dates and kinds of different treatment lines, as well as dates of follow-up and death were documented in chart review. Date of progression, including locoregional and distant metastatic progression, was reviewed in radiologists reports or physician notes. Date of death was matched with Swiss registry of deaths.

### Disease characteristics

GEP-NETs were classified according to TNM staging system of ENETS. Stage I and II were combined in localized disease, Stage III named as regional and Stage IV as distant disease or both of them combined in advanced disease [11]. We used ENETS/WHO 2010 grading system for classification into different grades according to proliferation index Ki-67 and mitotic count (10 HPF). (Appendix A2) [8, 11].

Treatment characteristics and follow-up data can be found in the supplementary material of this manuscript. (Appendix A3).

### Primary endpoint and outcome

For final analysis, we calculated PFS for each treatment as primary endpoint and for secondary endpoints we analysed overall survival (OS) and time to progression (TTP), all of them in months. (Appendix A4) [39, 40].

Patients who had received at least one therapy line were included in statistical analysis of treatment characteristics and PFS. PFS was calculated for implemented treatments and their sequences (Appendix A5) [41, 42].

### Statistics

All analyses were performed in IBM SPSS Statistics, version 24 (IBM Corp., Armonk, NY, USA). Descriptive statistics were used to calculate proportions and frequencies for categorical variables, means, medians and 95% confidence intervals (CI’s) for continuous variables. We used Kaplan Meier curves to evaluate OS, TTP and PFS rates. We compared these by log-rank test and used Kaplan Meier estimator as well as Cox regression to compare survival rates between different sites of localization, grades or stages at first diagnosis and treatment patterns [43] (Appendix A6) [41, 42]. The level of significance was set at *p* < 0.05. No multiple testing correction was adjusted to the *p*-values.

### Ethics

An application for this study was submitted to Swiss Ethics for human research and was adopted by Swiss Ethics Committees in 2016. (Appendix A7) [44].

## Results

Table 1 describes our patient population including patients’, disease characteristics, diagnostics and treatment characteristics.

**Table 1:** Overview of patients’, disease characteristics, diagnostics and treatment characteristics

**Patients’ characteristics**	**No. of patients (%)**
Male	136 (53%)
Female	120 (47%)
BMI, kg/m^2^	
< 18.5	8 (3%)
18.5 – 24.99	61 (24%)
25 – 29.99	40 (16%)
> 30	16 (6%)
	Mean, ± SD (range)
Age, years	55.6, ± 15.36 (13 – 86)
**Disease characteristics**	**No. of patients (%)**
Site of GEP-NET	
Small intestine	93 (36%)
Pancreas	88 (35%)
Appendix	19 (7.5%)
Stomach	16 (6%)
Rectum	11 (4%)
Colon or Cecum	7 (3%)
Gallbladder	1 (0.5%)
Unknown primary site	21 (8%)
Stage (ENETS)	
I	33 (13%)
II	32 (12%)
III	57 (22%)
IV	101 (40%)
Unknown	33 (13%)
Grade (ENETS/WHO 2010)	
G1	80 (31%)
G2	75 (29%)
G3	25 (10%)
Unknown	76 (30%)
Number of metastatic sites	
0	122 (48%)
1	69 (27%)
2	44 (17%)
Metastatic sites	
Lymph node	139 (54%)
At first diagnosis	50 (37%)
At distant stage	10 (8%)
At first diagnosis and at distant stage	74 (55%)
Liver	117 (46%)
Bone	45 (18%)
Peritoneal	29 (11%)
Lung	12 (5%)
Symptoms	
Pain	97 (38%)
Diarrhea	31 (12%)
Nausea/Vomitus	23 (9%)
Flush	20 (8%)
Weight loss	16 (6%)
Constipation	8 (3%)
Fatigue	6 (2%)
Acid Reflux/Gastritis	5 (2%)
Hypoglycaemia	3 (1%)
Hypertension	3 (1%)
**Diagnostic imaging**	
^68^ Ga-DOTATATE-PET-CT	188 (73%)
At first diagnosis	101 (54%)
At metastatic disease	63 (33%)
At first diagnosis and at metastatic disease	24 (13%)
CT	167 (65%)
MRI	89 (35%)
**Treatment characteristics**	**No. of patients (%)**
Kind of therapy (all subsequent lines)	
Surgery	172 (67%)
R0 resection	91 (53%)
SSA (Octreotide, Lanreotide, Pasireotide)	77 (30%)
PRRT	57 (22%)
Chemotherapy	40 (16%)
Endoscopic resection	29 (11%)
SIRT	25 (10%)
Everolimus	23 (9%)
Watch and wait	18 (7%)
Radiotherapy	13 (5%)
Sunitinib	7 (3%)
RFA	7 (3%)
TA(C)E	7 (3%)

For the sections “disease characteristics” (esp. metastatic sites and symptoms), “diagnostic imaging” and “treatment characteristics” multiple options per patient are possible

Abbreviations: ^68^* Ga* Gallium, *BMI* Body mass index, *CT* Computed tomography, *ENETS* European neuroendocrine tumor society. *G1 – G3* Grade 1 – Grade 3, *GEP-NET* gastroenteropancreatic neuroendocrine tumor, *MRI* Magnetic resonance imaging, *PRRT* Peptide receptor radionucleotide therapy, *RFA* Radiofrequency ablation, *SIRT* Selective internal radiation therapy, *SSA* Somatostatin analogues, *TA(C)E* Transarterial (chemo-) embolization, *WHO* World Health Organization

### Patients’ characteristics

Out of 328 NET patients between the age of 8 and 86 years (at first diagnosis), 256 patients with a GEP-NET were screened and included in analysis. At the beginning of diagnosis two children with GEP-NET were at age of 13 and 15. All GEP-NET patients have reached adult age at the end of observation period.

Only 26% were cancer care patients and underwent diagnostics, therapy and follow-up at USZ. 24% went through specific therapy at our institution, 41% were sent for a specific diagnostic and 9% were just presented at the MTB for second opinion. The average follow-up time was 77 months (range, 0.5 – 291 months). {Appendix A8} 14 patients suffered from a secondary malignancy during time of observation.

### Disease characteristics

A detailed overview of subgroups by origin and grading of our GEP-NET/(NEC) population is depicted in Table 1.

55% patients presented with NET-associated symptoms. Interestingly, the majority (58%) were symptomatic only at first diagnosis, while in 3% of cases, the symptoms were exclusively caused by metastases. We detected 10% of NEC G3, G1 and G2 were equally presented with 29–31% in our population. Most primary tumors were detected in pancreas (35%) and small intestine (36%), whereas one fourth of our GEP-NETs has been detected at a localized stage and 40% at metastatic stage. Within metastatic stage, liver involvement (46%), bone (18%) and peritoneal metastasis (11%) were the most common detected regions.

Patients with p-NET, NET of small intestine (s-NET) and of unknown primary site were commonly diagnosed in advanced stage and suffered significantly more from distant metastases than patients with NET of other localizations (*p* < 0.001) (Appendix A9).

### Treatment characteristics

**Fig. 1:**
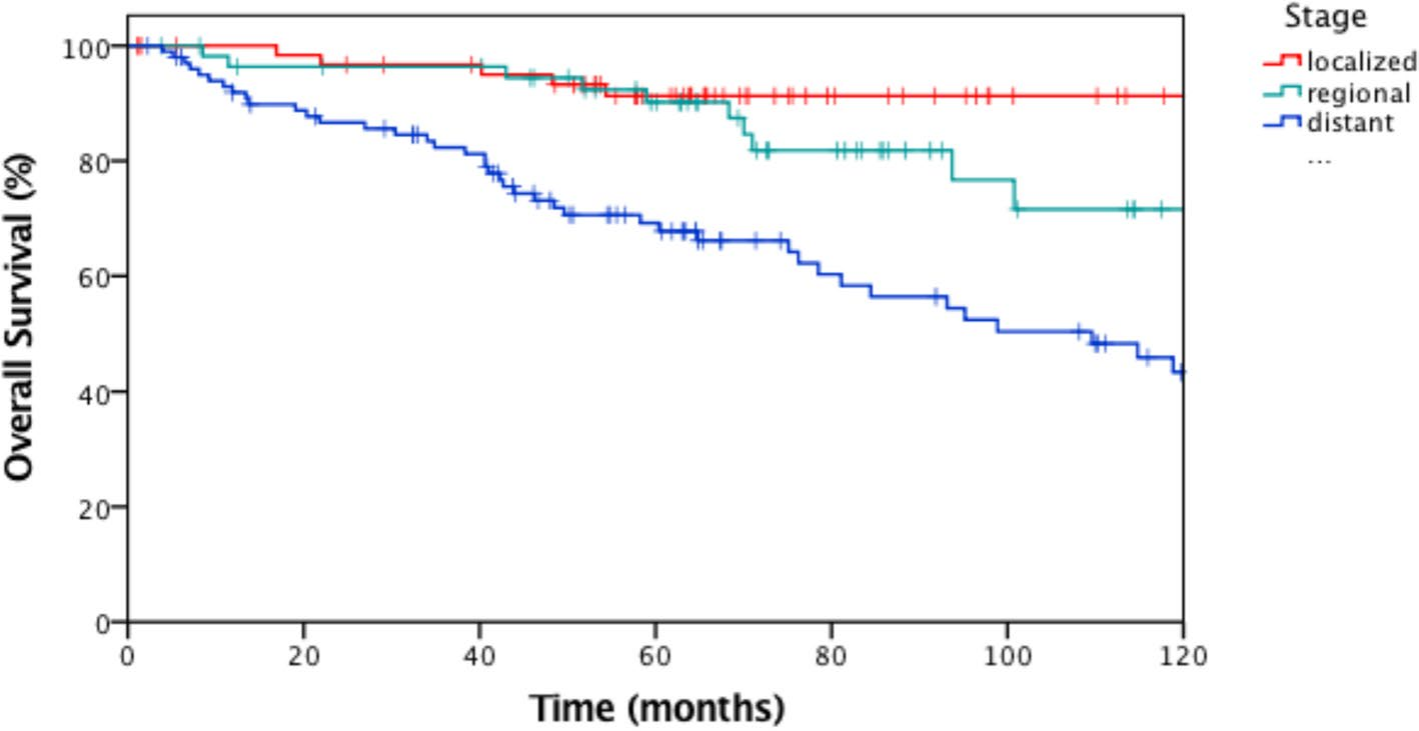
Treatment sequences showed by Sankey diagram [41, 33, 34]. Notes: 12 patients without any documented treatment were excluded from treatment pattern analysis. Lines ending without declarations of treatment represent patients who were not treated any further. Multiple treatment options per patient are possible. Abbreviations: CTx, chemotherapy; ER, endoscopic resection; LDT, liver directed therapy (radiofrequency ablation [RFA], selective internal radiation therapy [SIRT], transarterial [chemo-] embolization [TA(C)E]); mTOR, mTOR-inhibitors; PRRT, peptide receptor radionucleotide therapy; RT, radiotherapy; SSA, somatostatin analogues; SX, surgery; TKI, tyrosine kinase inhibitor; WW, watchful waiting

Two hundred forty-four patients underwent at least one therapy line and all patients had been discussed at least once at our MTB. In our study population a patient received averagely 2.6 different treatment sequences (range 1 to maximum of 13 therapies). The amount of therapy sequences increased significantly with higher stages (*p* < *0.001*) and higher grades (*p* = *0.004)* at first diagnosis. A Sankey diagram (Fig. 1) shows different treatment sequences. (Appendix A10).

Surgery was in general the most abundant therapy with a total of 233 surgical operations in 172 (67%) patients and by far the most performed first-line therapy. 53% of all surgeries were reported as R0 resected. Advanced GEP-NETs underwent a higher number of surgical operations than patients with locoregional disease, while these underwent surgery more frequently as a solitary treatment.

30% of GEP-NETs underwent therapy with SSA, mostly received during first-line setting (Appendix A11) [45, 46].

Relatively few patients received targeted therapies like mTOR (Everolimus [9%]) or tyrosine kinase inhibitors (Sunitinib, [3%]). These treatments were only used in advanced disease, higher grading of G2 and mostly as third-line or subsequent therapy.

PRRT for GEP-NETs has been commonly used in our population. 22% patients were treated at least once with PRRT. 44 (77%) of them suffered from distant disease at time of first diagnosis (Appendix A12).

16% of patients were treated with chemotherapy, and pancreatic NETs (p-NETs) as well NETs of unknown origin received more often classical chemotherapy than well-differentiated extra-pancreatic NETs in their medical history. 18 patients were treated more than once with at least one of these agents (either CAPTEM, Carboplatin/Cisplatin and Etoposid or STZ plus 5-FU), whereas 22% resulted in first- or second-line treatment. CAPTEM was mostly used in G2 NETs. The combination of Carbo- or Cisplatin with Etoposide was mostly given in G3 NETs/NEC or as a subsequent treatment in G2 NETs. Chemotherapy with Streptozotocin/5-FU was only implemented in G2 NETs.

### Secondary endpoints and outcome

Median OS of all patients was 181 months (95% CI, 106 – 256 months). The OS curves differed significantly between different sites of GEP-NET (*p* < *0.001*). The longest median OS was observed in p-NET patients (251 months; 95% CI, 104 – 399 months), the shortest median OS in patients with NET of unknown primary site (78.5 months; 95% CI, 1 – 156 months), (HR = 0.40; 95% CI, 0.20 – 0.80; *p* = *0.009*).

**Fig. 2:**
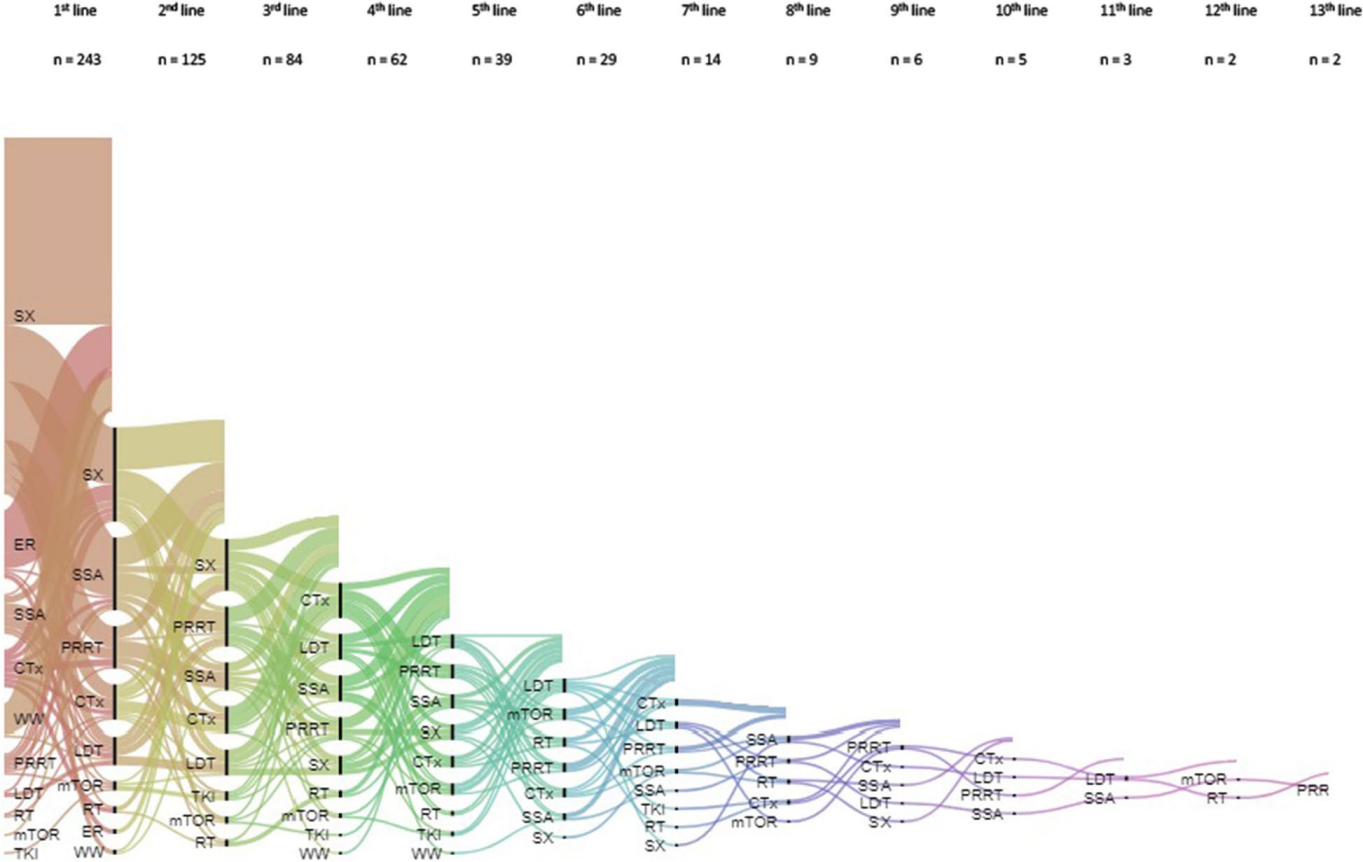
Kaplan Meier curves showing OS (months) according to different stages (ENETS) at first diagnosis, log-rank test, *p* < 0.001. Notes: Marks indicate censored cases. Median OS over all patients was 181 months (95% CI, 106 – 256 months). Abbreviations: CI, confidence interval; ENETS, European Neuroendocrine Tumor Society; OS, overall survival

The OS distributions also differed between stages (*p* < *0.001*), ((Fig. 2) and grades. While in localized disease (Stage II) the median OS was 144 months (95% CI, 116 – 172 months), in distant disease (Stage IV) we observed a median OS of 109 months (95% CI, 77 – 142 months), (HR = 0.31; 95% CI, 0.12 – 0.79; *p* = *0.01*). In G1 GEP-NETs, median OS was 139 months (95% CI, 126 – 152 months), in moderately differentiated disease (G2) we observed a median OS of 114 months (95% CI, 77 – 153 months), while in G3 GEP-NETs or -NECs median OS was with 40 months (95% CI, 7 – 73 months) shorter (G1 versus G3; HR = 0.27; 95% CI, 0.13 – 0.54; *p* < *0.001*; G2 versus G3; HR = 0.45; 95% CI, 0.23 – 0.88; *p* = *0.02*) (Appendix A13).

Median TTP of all patients was 84 months (95% CI, 52 – 116 months). TTP curves differed also between different sites of GEP-NET (log-rank test, *p* < *0.001*), with an extended median TTP from p-NETs of 107 months (95% CI, 64 – 150 months), while for a NET of unknown primary site resulted in the shortest median TTP of 34 months (95% CI, 0 – 73 months) was observed (HR = 0.33; 95% CI, 0.19 – 0.59; *p* < *0.001*).

TTP differed between different grades and stages at time of first diagnosis (both, log-rank test, *p* < *0.001*). We observed a significantly longer median TTP for Stage III-GEP-NETs with 109 months (95% CI, 72 – 146 months) than for Stage IV-GEP-NETs of 23 months (95% CI, 14 – 31 months; HR = 0.27; 95% CI, 0.17 – 0.44; *p* < *0.001*) (Appendix A14).

### Influence of treatments and their sequences to progression free survival

Patients who underwent systemic treatment or were treated with SSA showed negative outcome (shorter PFS) after a following operative intervention (systemic treatment; HR = 3.44; 95% CI, 1.85 – 6.38; *p* < *0.001;* SSA; HR = 3.41; 95% CI, 2.09 – 5.56; *p* < *0.001*).

SSA-related PFS was shorter if patients were additionally treated with systemic treatment (HR = 2.05; 95% CI, 1.12 – 3.78; *p* = *0.02*). PFS of PRRT was shorter if patients underwent systemic treatment during time of disease (HR = 2.02; 95% CI, 1.02 – 4.01; *p* = *0.04*).

Patients undergoing non-surgical interventions like radio-frequency ablation (RFA), selective internal radiotherapy (SIRT), radiotherapy or transarterial (chemo)-embolization (TA(C)E) had a shorter PFS after systemic treatments (HR = 3.35; 95% CI, 1.27 – 8.84; *p* = *0.02*)*.*

In Stage IV, we found a positive effect of PFS after treatment with SSA (HR = 0.45; 95% CI, 0.21 – 0.97; *p* = *0.04*) and systemic treatment (HR, 0.51; 95% CI, 0.26 – 0.99; *p* = *0.047*) if patients underwent prior surgery or endoscopic resection.

In G2 NETs, treatment with SSA showed a longer PFS if patients were also treated with a non-surgical intervention (RFA, SIRT, radiotherapy or TA(C)E), (HR, 0.33; 95% CI, 0.12 – 0.97; *p* = *0.04*). On the other hand, in G1 NETs we found shorter PFS of patients undergoing this treatment sequence (HR, 3.52; 95% CI, 1.05 – 11.8; *p* = *0.04*).

In patients with G1 and G2 NETs, shorter PFS after operative interventions was shown, if patients were treated with prior PRRT (G1 NETs; HR = 3.06; 95% CI, 1.19 – 7.87; *p* = *0.02*; G2 NETs; HR = 3.12; 95% CI, 1.17 – 8.29; *p* = *0.02*).

## Discussion

In this real-life study at an ENETS CoE, we succeeded in evaluating efficacy of treatment (PFS) according to primary site, stage and grade of tumor [3, 47]. To the best of our knowledge, this is the first analysis in this field with focus on long treatment sequences of GEP-NETs as well as on subanalyses of different types of GEP-NETs and multi-sequence treatments. There have been other studies that also focused on treatment patterns in GEP-NETs, but rather on fewer treatments and did not differ between various stages, grades or sites of neoplasm, which is relevant for daily clinical practice [39, 48].

Most studies exploring this rare and heterogeneous tumors are placebo-controlled intervention studies exploring a single treatment. The comparison of different treatment options in this real-life population study therefore is a valuable addition and helps clinical decision making [27, 32, 49] (Appendix A15).

In our data collection, most patients were of normal weight or overweight. There are studies, which have observed that medications (mTOR-Inhibitors or PRRT) are not ideally calculated according to BMI [50–52].

Frequency and survival rates of different primary sites differed remarkably compared to well-known prevalence and outcome of GEP-NETs [6]. In our cohort, rectal NETs (4%) and NETs of colon (3%) are underrepresented in comparison to generally known epidemiological data in US population (26 – 34% rectum, 16 – 18% colon), whereas pancreatic NETs with 35% are overrepresented (12% in US population) [8, 53, 54]. One of the reasons might be, that our data was collected in a ENETS CoE, respectively a tertiary hospital (Appendix A16).

**Fig. 3:**
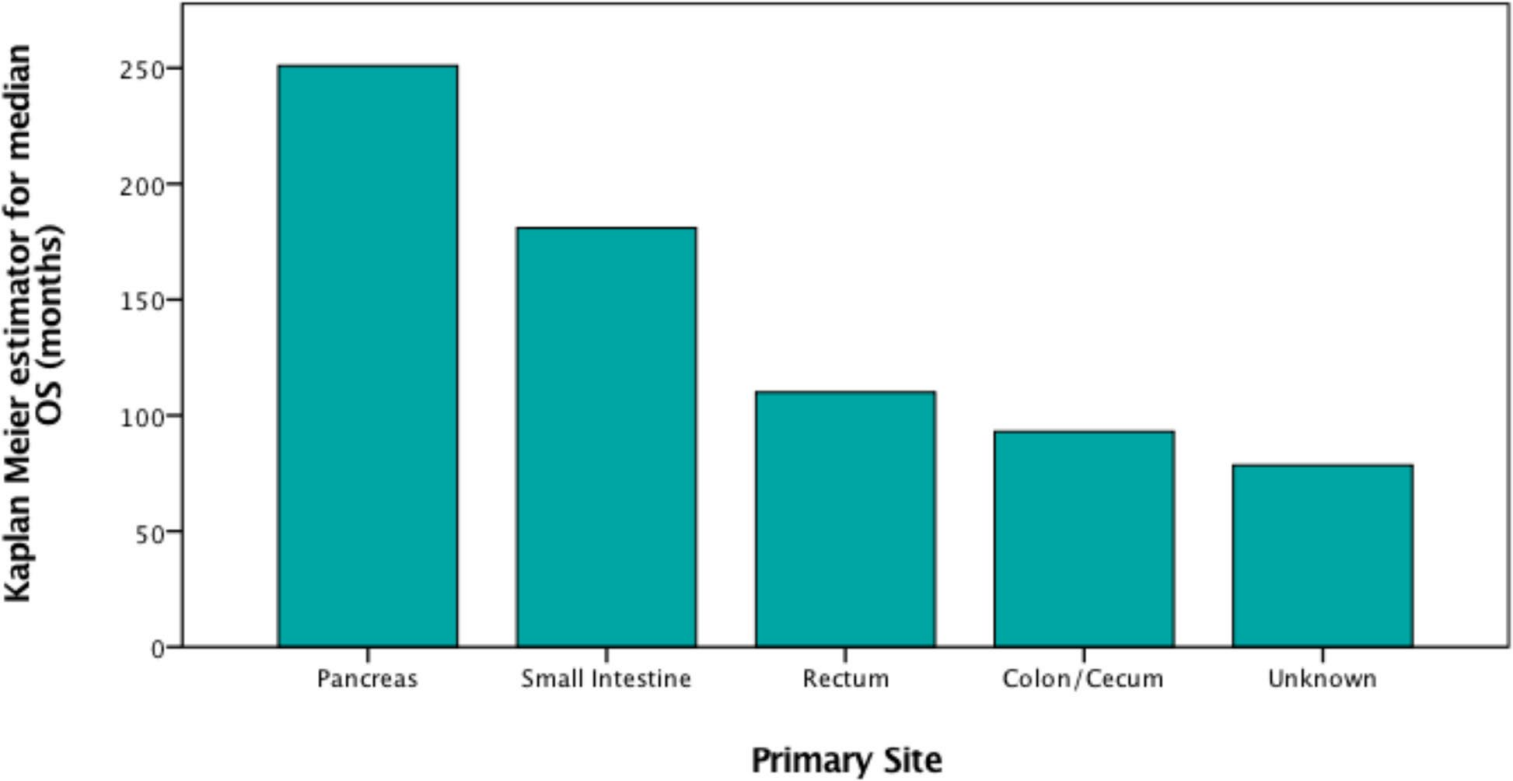
Kaplan Meier estimator for median OS of GEP-NET patients according to primary site [6]. Notes: We were not able to investigate median OS of gastric NET patients and patients with appendix NET, due to the fact that less than 50% of these patients died until the end of the observation period. The single patient with NET of the gallbladder was extracted from illustration due to lack of statistical power. Median OS over all patients was 181 months (95% CI, 106 – 256 months). Abbreviations: CI, confidence interval; GEP, gastroenteropancreatic; NET, neuroendocrine tumor; OS, overall survival

Against expectations, the longest median OS of our population was seen in p-NET patients (Fig. 3), even though p-NET patients were overrepresented in the reported NET-related deaths. One possible explanation, why p-NETs in our study achieved a much better outcome than other primary sites might be the higher presentation in localized stages in comparison to other sites (31% versus 18% in rectal NET), (Table B1) [6] (Appendix A17) [3, 11, 24, 55].

Our analysis used pathologic reports before the updated WHO 2017 classification, whereas separation of G3 NET and G3 NEC was not made as well we could not translate our data to the updated WHO 2019 classification [13]. Plus, no additional pathologic features on high grade GEP-NEN and GEP-NEC, which have nowadays an impact for systemic treatment choices as Platinum and Etoposide or CAPTEM based regimens were available and as represented now in the updated ESMO 2020 guidelines [17, 56].

Our study showed more surgical interventions in advanced setting than in localized disease in contrary to our expectation. The overrepresentation of surgical interventions in this subgroup is most probably based on metastatic resection, liver surgery combined with liver directed therapy (SIRT, RFA, TA(C)E) or urgent surgery, since advanced disease commonly makes local complications like intestinal obstruction or bleeding. However, we were able to demonstrate a benefit of treatment-adjusted PFS (especially for SSA and systemic therapies) in Stage IV if patients underwent prior surgical intervention, against expectations and in contrast to other literature [55] (Appendix A18), (Appendix A19), (Appendix A20) [3, 11, 25, 39, 55, 57, 58].

On the contrary, in highly selected cases the choice for the right timing of liver surgery in oligometastatic GEP-NETs improves survival and the liver-directed strategy should be discussed among an expert team in NET and liver surgery, mostly placed at ENETS CoE [26, 59, 60]. In advanced disease, we investigated a benefit of PFS after treatment with SSA and systemic treatment, if patients underwent prior surgery or endoscopic resection. Patients with initial operative treatment in distant disease underwent probably a cytoreductive treatment or were generally in a high performance status before undergoing surgical interventions, which concludes in longer PFS after subsequent treatments. These results are very encouraging for the role of surgery in advanced GEP-NET and should be evaluated in future clinical trials.

In G2 NETs, treatment with SSA showed longer PFS if patients were also treated with a non-surgical intervention (RFA, SIRT, Radiotherapy or TA(C)E).

In our study, G1 and G2 NETs showed shorter PFS after surgical interventions if they were treated with prior PRRT. But independently from surgical interventions, PRRT may affect outcome in a positive way if using it in an early phase of treatment sequence. This is currently investigated in clinical trials [61, 62].

Given the character of this retrospective study with multiple potential biases, care must be taken to apply these results for clinical practice [63].

**Fig. 4:**
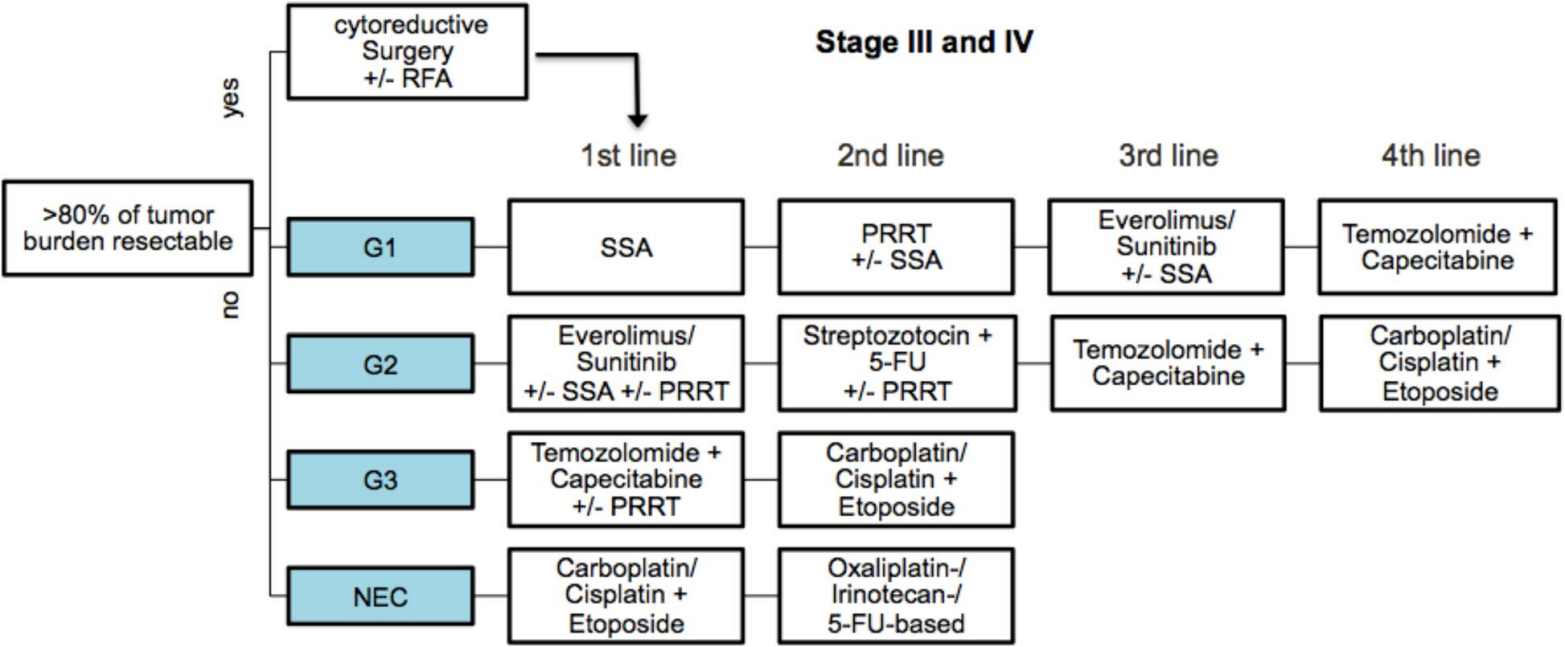
Flow chart shows suggested treatment algorithm for advanced GEP-NETs (Stage III and IV, ENETS) according to the finding in our study and recommendations of other studies [3, 11, 24, 35, 40]. Abbreviations: 5-FU, 5-Fluorouracil, ENETS, European Neuroendocrine Tumor Society; G1 – G3, grade 1 – grade 3; GEP-NETs, gastroenteropancreatic neuroendocrine tumor; NEC, neuroendocrine carcinoma; PRRT, peptide receptor radionucleotide therapy; RFA, radiofrequency ablation; SSA, somatostatin analogues

According to the findings based on our results, we suggest following treatment algorithm (Fig. 4) for advanced GEP-NETs [3, 11, 24, 55, 57]. Interestingly, our recommendation in Fig. 4 for our patient collective of a period from 2012–2016 is in line with the recent recommendation by ESMO and NCCN for GEP-NETs [17, 31]. Again, predictive biomarkers for GEP-NETs are quite limited and remain still to origin of the primary, grading and SSTR2 positivity. For sure, whole genome sequencing, as the study by Scarpa et al. revealed highly interesting data for pancreatic NET for diagnosis and mostly for prediction. Some analysis even directed to the prediction of mTOR pathway or MGMT status in favour for CAPTEM. But this has to be proven within clinical trial and these biomarkers have been again highly discussed among the annual ENETS conference in 2022 [64, 65].

A demanding and unmet need will be to characterize patients in the planned course of systemic treatment by chemotherapy, for example the CAPTEM combination [66, 67]. Therefore, options either to treat by fixed courses or continuous treatment until progression are possible. However, our study was underpowered in numbers and subtypes of GEP-NET to give an insight within our patient population to this dedicated and demanding field. This should be focused on upcoming clinical trials.

Another fact to this treatment scenarios compared to the latest guidelines still is the lack of new treatment options within the last years. Promising results of the DUNE trial for G3 NET and NEC reflected a trend in survival for the checkpoint blockade combination durvalumab and tremilimumab and new TKI as surufatinib in the SANET and SANET-p trial demonstrated significant improvements in GEP-NETs with advanced staged. But these therapies did not receive an EMA or SwissMedic approvement so far [68–70].

### Strength and limitation of the study

Although USZ was at the time of study analysis the only certified ENETS CoE in Switzerland, the cohort of this study contains of a relatively small study population that induces limited explanatory power in statistical analyses. Nevertheless, in the present study a great overview of all patients’ disease and treatment characteristics, as well as survival rates at our center over the last years was shown. Due to this, it is now possible to compare this data collection of GEP-NETs at USZ with those of other international ENETS CoE within the planned registry of the ENETS group in the future.

The main limitation of this study is the retrospective nature, especially regarding the comparison of PFS after different treatments [63]. Further, data of our patients was collected within clinical routine, which may cause additional biases and delimitates the explanatory power of our results.

Thus, we lack of information for all completed treatment lines or transformation into higher grade in progression of disease, which resulted in an incomplete statement about treatment-decisions in our population.

A detailed statement of further minor limitation is placed at the appendix of study (Appendix A21) [3, 11, 58].

Based on our data, the best survival for a specific treatment sequence cannot be defined, since an insufficient number of patients received identical treatment sequences in this relatively small cohort. Above-noted treatment algorithm has been matched with recommendations of other studies [3, 11, 24, 55, 57] (Appendix A22) [11, 57, 58].

## Conclusions

Treatment with SSA is still the primary choice of first-line therapy in well-differentiated GEP-NETs (G1). PRRT gained importance as an alternative or combination option to other treatments in low (G1) or intermediate grade (G2) GEP-NETs and may affect outcome in a positive way if using it in an early phase of treatment sequence [61, 71]. Inhibitors of mTOR or tyrosine kinase (in this cohort everolimus or sunitinib) alone or in combination with SSA are considered the best third-line treatment in G1 NETs or in first-line therapy in G2 NETs, also in combination with PRRT. Despite all these emerging treatments, we are still reliant upon systemic and cytostatic chemotherapy for treating high-grade GEP-NETs (G3 or -NECs).

With this study we expect to build a platform for future investigation and for developing future prospective and randomized studies of treatment sequences for GEP-NETs.

## Supplementary Information

**Additional file 1: Appendix Table B1.** Stages (ENETS) atinitial diagnosis according to primary site. **Table B2.** Different treatments(all subsequent lines) according to initial grade (ENETS/WHO 2010) of disease. **Figure B1.**Kaplan Meier curves showing OS (months) according to different grades(ENETS/WHO 2010) at first diagnosis, log-rank test, *p* = 0.001. **Figure B2.**Kaplan Meier estimator for median OS of GEP-NET patients according to primarysite and grade (ENETS/WHO 2010).

## Data Availability

The datasets used and/or analysed during the current study are available from the corresponding author on reasonable request*.*

## References

[1] Rossi RE, Ciafardini C, Sciola V, Conte D, Massironi S (2018). Chromogranin A in the Follow-up of Gastroenteropancreatic Neuroendocrine Neoplasms. Pancreas.

[2] Kos-Kudła B, Čwikła J, Ruchała M (2017). Current treatment options for gastroenteropancreatic neuroendocrine tumors with a focus on the role of lanreotide. Wspolczesna Onkol.

[3] Öberg K (2012). Neuroendocrine tumors of the digestive tract: Impact of new classifications and new agents on therapeutic approaches. Curr Opin Oncol.

[4] Oncology P. Database Report 2015. 2016. https://www.swissnet.net/images/files/SwissNETReport2015.pdf.

[5] Kollar A, Bütikofer L, Ochsenbein A, Stettler C, Trepp R. Treatment sequence in patients with neuroendocrine tumours: a nationwide multicentre, observational analysis of the Swiss neuroendocrine tumour registry. Swiss Med Wkly. 2020;150:w20176. 10.4414/smw.2020.20176. 10.4414/smw.2020.20176 31940430

[6] Dasari A, Shen C, Halperin D (2017). Trends in the incidence, prevalence, and survival outcomes in patients with neuroendocrine tumors in the United States. JAMA Oncol.

[7] Huguet I, Grossman AB, O’Toole D (2016). Changes in the Epidemiology of Neuroendocrine Tumours. Neuroendocrinology.

[8] Kim JY, Hong SM (2016). Recent updates on neuroendocrine tumors from the gastrointestinal and pancreatobiliary tracts. Arch Pathol Lab Med.

[9] Pape UF, Jann H, Müller-Nordhorn J (2008). Prognostic relevance of a novel TNM classification system for upper gastroenteropancreatic neuroendocrine tumors. Cancer.

[10] Hochwald SN, Zee S, Conlon KC (2002). Prognostic factors in pancreatic endocrine neoplasms: An analysis of 136 cases with a proposal for low-grade and intermediate-grade groups. J Clin Oncol.

[11] Öberg K, Knigge U, Kwekkeboom D, Perren A. Neuroendocrine gastro-entero-pancreatic tumors: ESMO clinical practice guidelines for diagnosis, treatment and follow-up. Ann Oncol. 2012;23(SUPPL. 7). 10.1093/annonc/mds295. 10.1093/annonc/mds295 22997445

[12] Kim JY, Hong SM, Ro JY (2017). Recent updates on grading and classification of neuroendocrine tumors. Ann Diagn Pathol.

[13] Assarzadegan N, Montgomery E (2021). What is new in the 2019 world health organization (WHO) classification of tumors of the digestive system: Review of selected updates on neuroendocrine neoplasms, appendiceal tumors, and molecular testing. Arch Pathol Lab Med.

[14] Kung CH, Ou JL, Chen BC, Lin JC (2018). Gastrointestinal neuroendocrine tumors. J Intern Med Taiwan.

[15] Öberg K (2017). Medical Therapy of Gastrointestinal Neuroendocrine Tumors. Visc Med.

[16] Cavalcanti MS, Gönen M, Klimstra DS (2016). The ENETS/WHO grading system for neuroendocrine neoplasms of the gastroenteropancreatic system: a review of the current state, limitations and proposals for modifications. Int J Endocr Oncol.

[17] Pavel M, Öberg K, Falconi M (2020). Gastroenteropancreatic neuroendocrine neoplasms: ESMO Clinical Practice Guidelines for diagnosis, treatment and follow-up. Ann Oncol.

[18] Kaltsas G, Caplin M, Davies P (2017). ENETS Consensus Guidelines for the Standards of Care in Neuroendocrine Tumors: Pre- A nd Perioperative Therapy in Patients with Neuroendocrine Tumors. Neuroendocrinology.

[19] Partelli S, Maurizi A, Tamburrino D (2014). GEP-NETS update: A review on surgery of gastro-entero-pancreatic neuroendocrine tumors. Eur J Endocrinol.

[20] Pavel M, Valle JW, Eriksson B (2017). ENETS Consensus Guidelines for the Standards of Care in Neuroendocrine Neoplasms: Systemic Therapy-Biotherapy and Novel Targeted Agents. Neuroendocrinology.

[21] Garcia-Carbonero R, Rinke A, Valle JW, Fazio N, Caplin M, Gorbounova V, et al. PMACC participants. ENETS Consensus Guidelines for the Standards of Care in Neuroendocrine Neoplasms. Systemic Therapy 2: Chemotherapy. Neuroendocrinology. 2017;105(3):281. 10.1159/000473892. 10.1159/000473892 28380493

[22] Hicks RJ, Kwekkeboom DJ, Krenning E, Bodei L, Grozinsky-Glasberg S, Arnold R, et al. RJACC participants. ENETS Consensus Guidelines for the Standards of Care in Neuroendocrine Neoplasia: Peptide Receptor Radionuclide Therapy with Radiolabeled Somatostatin Analogues. Neuroendocrinology. 2017;105(3):295. 10.1159/000475526. 10.1159/000475526 28402980

[23] Knigge U, Capdevila J, Bartsch DK, Baudin E, Falkerby J, Kianmanesh R, et al. VMACCPACC participants. E. NETS Consensus Recommendations for the Standards of Care in Neuroendocrine Neoplasms: Follow-Up and Documentation. Neuroendocrinology. 2017;105(3):310. 10.1159/000458155. 10.1159/000458155 28222443

[24] Uri I, Avniel-Polak S, Gross DJ, Grozinsky-Glasberg S. Update in the Therapy of Advanced Neuroendocrine Tumors. Curr Treat Options Oncol. 2017;18(12). 10.1007/s11864-017-0514-9 10.1007/s11864-017-0514-9 29143892

[25] Knigge U, Hansen CP (2012). Surgery for GEP-NETs. Best Pract Res Clin Gastroenterol.

[26] Siebenhüner AR, Langheinrich M, Friemel J, Schaefer N, Eshmuminov D, Lehmann K. Orchestrating Treatment Modalities in Metastatic Pancreatic Neuroendocrine Tumors — Need for a Conductor. 2022:1–17. 10.3390/cancers14061478 PMC8946777 35326628

[27] Caplin ME, Pavel M, Ćwikła JB (2016). Anti-tumour effects of lanreotide for pancreatic and intestinal neuroendocrine tumours: The CLARINET open-label extension study. Endocr Relat Cancer.

[28] Stueven AK, Kayser A, Wetz C, et al. Somatostatin analogues in the treatment of neuroendocrine tumors: Past, present and future. Int J Mol Sci. 2019;20(12). 10.3390/ijms20123049. 10.3390/ijms20123049 PMC6627451 31234481

[29] Castaño JP, Sundin A, Maecke HR (2014). Gastrointestinal neuroendocrine tumors (NETs): New diagnostic and therapeutic challenges. Cancer Metastasis Rev.

[30] Strosberg JR, Caplin ME, Kunz PL (2021). 177Lu-Dotatate plus long-acting octreotide versus high-dose long-acting octreotide in patients with midgut neuroendocrine tumours (NETTER-1): final overall survival and long-term safety results from an open-label, randomised, controlled, phase 3 trial. Lancet Oncol.

[31] NCCN Guidelines for Neuroendocrine and Adrenal Tumors. 2021.

[32] Pavel ME, Hainsworth JD, Baudin E (2011). Everolimus plus octreotide long-acting repeatable for the treatment of advanced neuroendocrine tumours associated with carcinoid syndrome (RADIANT-2): A randomised, placebo-controlled, phase 3 study. Lancet.

[33] Yao JC, Fazio N, Singh S, Buzzoni R, Carnaghi C, Wolin E, et al. Everolimus for the treatment of advanced, non-functional neuroendocrine tumours of the lung or gastrointestinal tract (RADIANT-4): a randomised, placebo-controlled, phase 3 study. Lancet. 2016;387(10022). 10.1016/S0140-6736(15)00817-X. 10.1016/S0140-6736(15)00817-X PMC6063317 26703889

[34] Fine RL, Gulati AP, Krantz BA (2013). Capecitabine and temozolomide (CAPTEM) for metastatic, well-differentiated neuroendocrine cancers: The Pancreas Center at Columbia University experience. Cancer Chemother Pharmacol.

[35] Raymond E, Dahan L, Raoul J-L, Bang Y-J, Borbath I, Lombard-Bohas C, et al. Sunitinib Malate for the Treatment of Pancreatic Neuroendocrine Tumors. N Engl J Med. 2011;365:687–696. 10.1056/NEJMoa1003825 21306237

[36] Clewemar Antonodimitrakis P, Sundin A, Wassberg C, Granberg D, Skogseid B, Eriksson B (2016). Streptozocin and 5-Fluorouracil for the Treatment of Pancreatic Neuroendocrine Tumors: Efficacy. Prognostic Factors and Toxicity Neuroendocrinology.

[37] Shah MH, Goldner WS, Halfdanarson TR, et al. Neuroendocrine and adrenal tumors, version 2.2018 featured updates to the nccn guidelines. JNCCN J Natl Compr Cancer Netw. 2018;16(6):693–702. 10.6004/jnccn.2018.0056. 10.6004/jnccn.2018.0056 29891520

[38] Krug S, Damm M, Garbe J, et al. Finding the appropriate therapeutic strategy in patients with neuroendocrine tumors of the pancreas: Guideline recommendations meet the clinical reality. J Clin Med. 2021;10(14). 10.3390/jcm10143023 10.3390/jcm10143023 PMC8304907 34300189

[39] Lurie RH, Cancer C, Group A, et al. Real-World Treatment Patterns and Clinical Outcomes in Advanced Gastrointestinal Neuroendocrine Tumors ( GI NET ): A Multicenter Retrospective Chart Review Study. 2019:1–10.10.1634/theoncologist.2018-0519 10.1634/theoncologist.2018-0519 PMC6693731 30606883

[40] Administration D (2007). Guidance for Industry: Clinical Trial Endpoints for the Approval of Cancer Drugs and Biologics. Biotechnol Law Rep.

[41] Prinja S, Gupta N, Verma R (2019). Censoring in Clinical Trials : Review of Survival Analysis Techniques Non-Parametric Survival Analysis : Kaplan Meier Product Limit Method.

[42] Vervölgyi E, Kromp M, Skipka G, Bender R, Kaiser T (2011). Reporting of loss to follow-up information in randomised controlled trials with time-to-event outcomes: A literature survey. BMC Med Res Methodol.

[43] van der Linden N, Bongers ML, Coupé VMH (2017). Treatment Patterns and Differences in Survival of Non-Small Cell Lung Cancer Patients Between Academic and Non-Academic Hospitals in the Netherlands. Clin Lung Cancer.

[44] Siebenhüner, Alexander (Oncology UHZ, Winder, Thomas (Oncology ALF. Protokoll für HFV/Ethikantrag Dissertation.pdf. 2016.

[45] Prostamed AV, Ag PR. Zugelassene Präparate / Médicaments autorisés. 2020.

[46] Swissmedic. 19.2.2019 Arzneimittelinformation. Fachinformation Somatuline Autogel 60 mg / 90 mg / 120 mg.

[47] Walenkamp A, Crespo G, Maya FF (2014). Hallmarks of gastrointestinal neuroendocrine tumours: Implications for treatment. Endocr Relat Cancer.

[48] Benson AB, Broder MS, Cai B, Chang E, Neary MP, Papoyan E (2017). Real-world treatment patterns of gastrointestinal neuroendocrine tumors: A claims database analysis. World J Gastroenterol.

[49] Yao JC, Pavel M, Lombard-Bohas C (2016). Everolimus for the treatment of advanced pancreatic neuroendocrine tumors: Overall survival and circulating biomarkers from the randomized, Phase III RADIANT-3 study. J Clin Oncol.

[50] Alsadik S, Gnanasegaran G, Chen L, et al. Single centre retrospective review of outcome of 177 Lu‐DOTATATE Peptide Receptor Radionuclide Therapy in treatment of progressive metastatic neuroendocrine tumors: survival, toxicity, and prognostic factors . J Neuroendocrinol. 2022;(June):1–9. 10.1111/jne.13210. 10.1111/jne.13210 36399420

[51] Ranallo N, Iamurri AP, Foca F, et al. Prognostic and Predictive Role of Body Composition in Metastatic Neuroendocrine Tumor Patients Treated with Everolimus: A Real-World Data Analysis. Cancers (Basel). 2022;14(13). 10.3390/cancers14133231. 10.3390/cancers14133231 PMC9264955 35805003

[52] Bellamy M, Chu B, Serencsits B (2022). Substantial External Dose Rate Variability Observed in a Cohort of LU-177 Patients Independent of Bmi and Sex. Radiat Prot Dosimetry.

[53] Tsikitis VL, Wertheim BC, Guerrero MA (2012). Trends of incidence and survival of gastrointestinal neuroendocrine tu-mors in the united states: A seer analysis. J Cancer.

[54] Cives M, Strosberg J. Gastroenteropancreatic neuroendocrine tumours. CA- a cancer J Clin. 2018:471–487. 10.3322/caac.21493. 10.3322/caac.21493 30295930

[55] Rinke A, Gress TM (2017). Neuroendocrine Cancer, Therapeutic Strategies in G3 Cancers. Digestion.

[56] Venizelos A, Elvebakken H, Perren A (2021). The molecular characteristics of high-grade gastroenteropancreatic neuroendocrine neoplasms. Endocr Relat Cancer.

[57] Neychev V, Kebebew E. Management Options for Advanced Low or Intermediate Grade Gastroenteropancreatic Neuroendocrine Tumors: Review of Recent Literature. Int J Surg Oncol. 2017;2017. 10.1155/2017/6424812. 10.1155/2017/6424812 PMC5448049 28593056

[58] Rinke A, Auernhammer CJ, Bodei L, et al. Treatment of advanced gastroenteropancreatic neuroendocrine neoplasia, are we on the way to personalised medicine? *Gut*. 2021;70(9):1768 LP - 1781. 10.1136/gutjnl-2020-321300 10.1136/gutjnl-2020-321300 33692095

[59] Clift AK, Kidd M, Bodei L, Toumpanakis C, Baum RP, Oberg K, Modlin IMFA (2020). Neuroendocrine Neoplasms of the Small Bowel and Pancreas. Neuroendocrinology.

[60] Frilling A, Modlin IM, Kidd M, Russell C, Breitenstein S, Salem R, Kwekkeboom D, et al. Recommendations for management of patients with neuroendocrine liver metastases. Lancet Oncol. 2014;15(1):e8–2. 10.1016/S1470-2045(13)70362-0. 10.1016/S1470-2045(13)70362-0 24384494

[61] Contacts S, Lin S. Efficacy and Safety of 177Lu-Dotatate PRRT in Metastatic GEP-NEN Patients. Case Med Res. 2020:1–7.

[62] Čwikła J. Peptide Receptor Radionuclide Therapy in the Treatment of Advanced, Non-resectable and/or Symptomatic Tumors With SSTR Overexpression. Case Med Res. 2019:1-12. 10.31525/ct1-nct04029428.

[63] Ezziddin S, Attassi M, Yong-Hing CJ (2014). Predictors of long-term outcome in patients with well-differentiated gastroenteropancreatic neuroendocrine tumors after peptide receptor radionuclide therapy with 177Lu-octreotate. J Nucl Med.

[64] Scarpa A, Chang DK, Nones K (2017). Whole-genome landscape of pancreatic neuroendocrine tumours. Nature.

[65] ENETS Conference Programme 2022. 2022.

[66] Bongiovanni A, Liverani C, Foca F (2021). Temozolomide Alone or Combined with Capecitabine for the Treatment of Metastatic Neuroendocrine Neoplasia: A “real-World” Data Analysis. Neuroendocrinology.

[67] Chatzellis E, Daskalakis K, Angelousi A, et al. Authors’ Response to the Letter by Lamarca et al. Entitled “temozolomide-Capecitabine Chemotherapy for Neuroendocrine Neoplasms: The Dilemma of Treatment Duration” Regarding "activity and Safety of Standard and Prolonged Capecitabine/Temozolomide Administ. Neuroendocrinology. 2020;110(1–2):158–160. 10.1159/000503999. 10.1159/000503999 31597137

[68] Hernando J, Manzano JL, Teule A, et al. DUNE trial. Ann Oncol. 2021;(32 (suppl_5)). doi:S906-S920. 10.1016

[69] Xu J, Shen L, Zhou Z, Li J, Bai C, Chi Y, Li Z, Xu N, Li E, Liu T, Bai Y, Yuan Y, Li X, Wang X, Chen J, Ying J, Yu X, Qin S, Yuan X, Zhang T, Deng Y, Xiu D, Cheng Y, Tao M, Jia R, Wang W, Li J, Fan S, Peng MSW (2020). Surufatinib in advanced extrapancreatic neuroendocrine tumours (SANET-ep): a randomised, double-blind, placebo-controlled, phase 3 study. Lancet Oncol.

[70] Xu J, Shen L, Bai C, Wang W, Li J, Yu X, et al. Surufatinib in advanced pancreatic neuroendocrine tumours (SANET-p): a randomised, double-blind, placebo-controlled, phase 3 study. Lancet Oncol. 2020;1489–1499. 10.1016/S1470-2045(20)30493-9. 10.1016/S1470-2045(20)30493-9 32966810

[71] Čwikła J. Peptide Receptor Radionuclide Therapy in the Treatment of Advanced, Non-resectable and/or Symptomatic Tumors With SSTR Overexpression. Case Med Res. 2019:1-13. 10.31525/ct1-nct04029428

