# Supplementary material for: What have we learnt from the past – would treatment decisions for GEP-NET patients differ between 2012 to 2016 by the new recommendations in 2022?
Source: BMC Cancer. 2023 Feb 13;23:148. doi: 10.1186/s12885-023-10567-1 (PMC9926660; doi:10.1186/s12885-023-10567-1)
Supplement: Supplementary file 1 — Additional file 1: Appendix Table B1. Stages (ENETS) atinitial diagnosis according to primary site. Table B2. Different treatments(all subsequent lines) according to initial grade (ENETS/WHO 2010) of disease. Figure B1.Kaplan Meier curves showing OS (months) according to different grades(ENETS/WHO 2010) at first diagnosis, log-rank test, p = 0.001. Figure B2.Kaplan Meier estimator for median OS of GEP-NET patients according to primarysite and grade (ENETS/WHO 2010). [file 12885_2023_10567_MOESM1_ESM.docx]

**Appendix A**

1. The retrospective observation period of these study patients was from date of initial diagnosis until December 2018, date of last contact or death.
2. G1 NET shows a proliferation (Ki-67) < 3%, G2 NET a Ki-67 index between 3% and 20% and NEC a proliferation index > 20%. In our analysis we were not able to consider the updated WHO 2017 or even WHO 2019 classification since neoplasm have been characterized through pathologists by the earlier WHO definition of 2010.
3. Treatments were pooled in different groups to investigate their influence on PFS. All surgical interventions including endoscopic resection were summarized as operative treatments; lanreotide and octreotide were summarized as SSA; chemotherapeutic therapies, as well as targeted treatments (Sunitinib and Everolimus) formed the group of systemic treatments. Several non-surgical interventions (RFA, SIRT, TA(C)E and radiotherapy) were combined. PRRT formed a group by itself, different PRRT sessions up to an interval of 8 to 10 weeks were reported as a single treatment line.
4. OS was defined as time from first diagnosis of disease to death of any cause. Similarly, NET-related OS was defined as time from first diagnosis of disease to NET-related death. As TTP we defined the time between date of first diagnosis and first objective progression (locally or metastatic). In case of death before progression, patients were censored at time of death. PFS for each treatment, was defined as time from treatment initiation to first objective tumor progression or NET-caused death (whichever comes first). In case of non-NET-related death, patients were censored at time of death.

### Patients with their latest documented reports (clinical information system) before December 2018 and without any further information on the course of their disease were defined as lost to follow-up. The respective data of these patients and patients without an event until the end of the observation period were classified as censored for survival analysis at time of last documented observation-date. All study endpoints were compared between different primary sites, stages and grades of NET.

1. The patients who were lost to follow-up were censored and therefore considered in the calculation of Kaplan Meier curves (including log-rank test) and Cox regression. Due to lack of statistical power, the single patient with NET of the gallbladder was extracted for survival analysis according to primary site. Patients without reported treatment were excluded from final analyses comparing PFS of treatment sequences.
2. Furthermore, included patients approved by written General Consent that their specific non-coded data was taken from KISIM CISTEC AG or patient files. This Data was then recorded as coded and anonymous data in chart review and used for statistical analysis.
3. 186 patients (73%) were censored for calculating OS, due to lost to follow-up or non-occurrence of death until the end of the observation period. 133 patients (52%) were censored for calculation of TTP, due to lost to follow-up or non-occurrence of progression. 115 patients (47%) were censored for calculation of PFS of all therapies, due to lost to follow-up, non-occurrence of NET-related death or progression.
4. We observed that appendix-NETs were diagnosed in locoregional disease and mostly as random diagnosis after appendectomy.
5. Patients with Stage I were treated with 1.2 therapy lines, Stage II- with 1.8, Stage III- with 2.2 and Stage IV-patients with 4 treatment lines on average. Patients with G1 GEP-NET underwent 2.3, G2 patients 2.8 and G3 patients 4 treatment lines on average.
6. In our cohort, lanreotide was just used between 2013 and 2018, which is caused by late marketing approval for treatment of GEP-NETs in 2016 in Switzerland. Octreotide was prescribed from 1994 to 2018.
7. PRRT was used 26 times as first- or second-line treatment (early PRRT), while 46 times PRRT was applied as third or subsequent therapy line (late PRRT).
8. Median NET-related OS was 251 months (95% CI, 188 – 314 months). 52 NET-related deaths were reported until the end of observation period. 21 (40%) of NET-related deaths were associated with p-NETs, while 13 (25%) were related to NETs of small intestine.
9. Well-differentiated (G1) GEP-NETs showed the longest median TTP with 122 months (95% CI, 83 – 161 months), while moderately differentiated (G2) GEP-NETs showed with 37 months (95% CI, 13 – 61 months) a shorter median TTP. G3 GEP-NETs showed the shortest median TTP of 12 months (95% CI, 7 – 16 months), (G1 versus G3; HR, 0.22; 95% CI, 0.13 – 0.38; p < 0.001; G2 versus G3; HR, 0.40; 95% CI, 0.24 – 0.67; p < 0.001).
10. In our center there is not only a single team responsible for GEP-NETs but rather these are separated into several departments with a dedicated ENETS responsible specialist (e.g., oncologist, gastroenterologist, surgeon, nuclear medicine, endocrinologist). Due to this, screening of patients, choice of diagnostics and treatment lines may differ between departments. Thus, up to now, only limited and heterogeneous data of GEP-NETs for ENETS CoEs exist.
11. Localized colorectal NETs, which were diagnosed and resected entirely by endoscopy, are rarely treated at our center. On the contrary, if colorectal NETs were notified at our center, they were referred due advanced stage for treatment making decisions. On the contrary, p-NETs even in early stages are referred to ENETS CoE in Switzerland as a multidisciplinary team with an experienced surgeon is mandatorily needed.
12. We focused especially on well- (G1) and moderately differentiated (G2) p-NETs and s-NETs, since these represented the largest subgroup of our population (46%). Most of these patients underwent surgery as first treatment line, followed by consecutive surgical intervention or treatment with SSA as second-line therapy. As third therapy line, PRRT was the most used treatment, followed by sunitinib as the second most common used agent. We observed a widespread diversity of treatments in fourth-line. Against our expectation SSA was the most frequently used treatment in fourth-line, followed by PRRT and chemotherapy. Prior treatment with SSA or PRRT didn’t show any effect on PFS after systemic treatment in G2 p-NET or s-NET (log-rank test, p = 0.96 for SSA; log-rank test, p = 0.50 for PRRT) against recommendations in common literature.
13. Patients who were treated with SSA, surgery or PRRT suffered from a shorter PFS, if they underwent prior systemic treatment. An explanation for this observation may be that patients, who were treated with systemic therapies in an early therapy phase mostly suffered from an aggressive disease, which was spread at time of diagnosis. For these patients, treatment strategies are difficult and consist in efforts to delay progression of disease instead of cure.
14. Outcome (PFS) of patients who underwent systemic therapy or treatment with SSA was shorter after a following operative intervention. One hypothesis of the reduced survival of surgery followed by systemic treatment may be the fact that these patients suffered from distant disease and underwent surgery to resect metastases or to solve complications as ileus within the metastatic setting. Since no distinction was made between different types of surgery when collecting this retrospective data, this assumption cannot be statistically confirmed and must be examined in future prospective studies.
15. Surgical intervention is, regarding to survival rates (PFS) in our population, a favourable initial treatment in any stage of disease and should be considered as cytoreductive treatment also in advanced disease to reduce symptoms and to improve better effect of following systemic therapies. In patients with advanced GEP-NET, resection as first-line treatment should only be considered, if over 80% of tumor burden can be safely resected. Well- or moderately differentiated GEP-NETs show a higher expression of SSTR, which is used for both diagnostic and therapy options. For this reason, SSA is primary choice of first-line treatment but only in well-differentiated GEP-NET, favourable due to its less toxic profile and high tolerance. If disease relapses, PRRT is considered as second-line treatment, which also makes use of a high expression of SSTR. mTOR or tyrosine kinase inhibitors (in our cohort Everolimus or Sunitinib) alone or in combination with SSA are considered the best third-line treatment. Fourth-line treatment consists of a chemotherapeutic approach with temozolomide in combination with capecitabine. Patients suffering from a G2 GEP-NET can be treated with chemotherapy with Streptozotocin and 5-FU alone or in combination with PRRT as second-line, if first-line treatment with mTOR or tyrosine kinase inhibitors alone, or in combination with SSA or PRRT has failed. As third-line therapy, another chemotherapeutic approach with temozolomide and capecitabine is considered. As fourth-line treatment, we recommend a platinum-based chemotherapy like carbo- or cisplatin in combination with etoposide.

In high-grade GEP-NETs (G3 or -NECs), the only suggested treatment with evidence of efficacy is chemotherapy. Subdivided into well-differentiated G3 NETs and poorly differentiated NECs according to WHO classification 2017, different chemotherapeutic agents are preferred. G3 GEP-NETs preferably are treated with temozolomide in combination with capecitabine as first choice and carbo- or cisplatin in combination with etoposide as second choice, while in NECs first choice consists in the latter. The only potential alternative treatment of NECs consists of a systemic chemotherapy based on oxaliplatin, irinotecan or 5-FU. Interestingly, our suggestion is in line with most recent treatment recommendation for personalized treatment approaches in G3 NEN and G3 NEC.

1. GEP-NET patients who were diagnosed before 2012 were included in statistical analysis only if they survived the starting point of screening phase in 2012. With dates of first diagnosis ranging back as far as 1986, less aggressive GEP-NETs may have had a higher chance reaching the screening period in 2012, while more severe courses of disease resulting in death before 2012 were not included in statistical analysis. This may result in a bias. Due to the high numbers of different treatments and disease subgroups, a substantial quantity of statistical tests was performed. However, no multiple test correction was made, therefore falsely significant results may have occurred. Hence the comparison to other studies is required. The broad variety of different treatment sequences led to small subgroups of patients who underwent a respective therapy sequence. Under these circumstances no evidence for a superior treatment sequence regarding survival rates was found. Nevertheless, we showed, like common recommendations, that the combination of surgery followed by SSA, PRRT and systemic treatment are favourable for survival (PFS). Last but not least, due the lack of biomarker information of our GEP-NET cohort we could not subcategorize our population based on the molecular class and match them to different treatment responses. A more precise characterization of GEP-NETs by different molecular features as vascular endothelial growth factor (VEGFR) for anti-VEGF targeting, program death ligand-1 (PD-1), mismatch repair deficiency (MMR-d) and combined positive score (CPS) for checkpoint-inhibitor targeting, O-6-methylguanine-DNA methyltransferase (MGMT) status for temozolomide efficacy as well evaluation of the RAS-RAF-MEK-ERK cascade inhibition for multikinase inhibitors as dabrafenib trametinib would be recommended to perform a personalized treatment profile for GEP-NET patients.

In future we aim to follow-up and monitor GEP-NET patients steadily and prospectively at our center to collect complete long-term data of a consistent GEP-NET cohort including an updated WHO 2019 grading system. In addition, collection of material (tissue, blood, stool, urine) for creating a unique biobank is planned.

1. We demonstrated that surgery, as initial and only curative treatment, is favourable regarding to survival rates. Cytoreductive surgery should be also considered for patients with advanced disease to reduce symptoms and to improve the effect of following systemic therapies.^11, 25^ However, tumor burden should be > 80% resectable.

**Appendix B**

No. of patients (%)

|  |  |  |  |  |  |  |
| --- | --- | --- | --- | --- | --- | --- |
| Primary Site | Stage I | Stage II | Stage III | Stage IV | Unknown | Total |
| Small intestine | 5 (5%) | 10 (11%) | 29 (31%) | 41 (44%) | 8 (9%) | 93 (36%) |
| Pancreas | 13 (15%) | 14 (16%) | 15 (17%) | 32 (36%) | 14 (16%) | 88 (35%) |
| Appendix | 7 (37%) | 6 (32%) | 2 (10%) | 3 (16%) | 1 (5%) | 19 (7.5%) |
| Stomach | 5 (31%) | 0 (0%) | 3 (19%) | 2 (12.5%) | 6 (37.5%) | 16 (6%) |
| Rectum | 2 (18%) | 0 (0%) | 3 (27%) | 2 (18%) | 4 (37%) | 11 (4%) |
| Colon or Cecum | 1 (14%) | 1 (14%) | 3 (43%) | 2 (29%) | 0 (0%) | 7 (3%) |
| Gallbladder | 0 (0%) | 1 (100%) | 0 (0%) | 0 (0%) | 0 (0%) | 1 (0.5%) |
| Unknown primary site | 0 (0%) | 0 (0%) | 2 (9%) | 19 (91%) | 0 (0%) | 21 (8%) |
| Total | 33 (13%) | 32 (12%) | 57 (22%) | 101 (40%) | 33 (13%) | 256 (100%) |

**Table B1:** Stages (ENETS) at initial diagnosis according to primary site.

Abbreviations: ENETS, European Neuroendocrine Tumor Society.

No. of patients

|  |  | |  | |  | |  | |  |
| --- | --- | --- | --- | --- | --- | --- | --- | --- | --- |
| Kind of treatment | G1 | G2 | | G3 | | Unknown | | Total | |
| Surgery | 68 | 52 | | 12 | | 40 | | 172 | |
| SSA (Octreotide, Lanreotide, Pasireotide) | 29 | 28 | | 7 | | 13 | | 77 | |
| PRRT | 14 | 19 | | 9 | | 15 | | 57 | |
| Chemotherapy | 3 | 19 | | 15 | | 3 | | 40 | |
| Endoscopic resection | 10 | 6 | | 1 | | 12 | | 29 | |
| SIRT | 6 | 9 | | 4 | | 6 | | 25 | |
| Everolimus | 6 | 10 | | 4 | | 3 | | 23 | |
| Watch and wait | 4 | 4 | | 0 | | 10 | | 18 | |
| Radiotherapy | 2 | 3 | | 4 | | 4 | | 13 | |
| Sunitinib | 2 | 4 | | 0 | | 1 | | 7 | |
| RFA | 2 | 1 | | 2 | | 2 | | 7 | |
| TACE | 4 | 2 | | 1 | | 0 | | 7 | |

**Table B2:** Different treatments (all subsequent lines) according to initial grade (ENETS/WHO 2010) of disease.

Notes: 12 patients without any documented treatment were excluded. Multiple treatment options per patient are possible.

Abbreviations: ENETS, European Neuroendocrine Tumor Society; G1 – G3, Grade 1 – Grade 3; PRRT, peptide receptor radionucleotide therapy; RFA, radiofrequency ablation; SIRT, selective internal radiation therapy; SSA, somatostatin analogues; TA(C)E, transarterial (chemo-) embolization; WHO, World Health Organization.


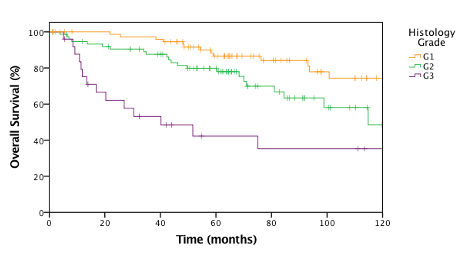


**Figure B1:** Kaplan Meier curves showing OS (months) according to different grades (ENETS/WHO 2010) at first diagnosis, log-rank test, p = 0.001.

Notes: Marks indicate censored cases. Median OS over all patients was 181 months (95% CI, 106 – 256 months).

Abbreviations: CI, confidence interval; ENETS, European Neuroendocrine Tumor Society; G1 – G3, Grade 1 – Grade 3; OS, overall survival; WHO, World Health Organization.


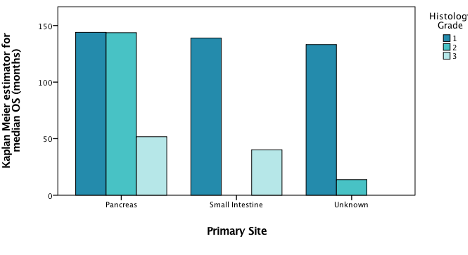


**Figure B2:** Kaplan Meier estimator for median OS of GEP-NET patients according to primary site and grade (ENETS/WHO 2010).^6^

Notes: 24 patients with a p-NET of unknown grade and a median OS of 265 months (95% CI, 243 – 288 months) were extracted from illustration. Due to low case numbers, we were not able to predict median OS for patients with G3 NET of unknown primary site and of patients with a NET of unknown primary site and unknown grade. We were also not able to predict median OS for patients with G2 NET of small intestine and those with a NET of small intestine of unknown grade, as well as NETs of several grades of other primary sites (gastric, rectal and appendix NET or NET of colon or cecum), due to the fact that in these subgroups less than 50% of the patients suffered from an event (death) until the end of the observation period.

The single patient with NET of the gallbladder was extracted from illustration due to lack of statistical power.

Median OS over all patients was 181 months (95% CI, 106 – 256 months).

Abbreviations: CI, confidence interval; ENETS, European Neuroendocrine Tumor Society; G1 – G3, Grade 1 – Grade 3; GEP, gastroenteropancreatic; NET, neuroendocrine tumor; OS, overall survival; WHO, World Health Organization.
